# Effect of exercise devised to reduce arm tremor in the sighting phase of archery

**DOI:** 10.1371/journal.pone.0285223

**Published:** 2023-05-01

**Authors:** Hiroshi Shinohara, Ryota Hosomi, Ryuji Sakamoto, Toshiya Urushihata, Shione Yamamoto, Chikashi Higa, Shinpei Oyama

**Affiliations:** 1 Department of Physical Therapy, Aomori University of Health and Welfare, Aomori, Japan; 2 Department of Physical Therapy, Ishikawa Hospital, Hyogo, Japan; 3 Department of Physical Therapy, Takarazuka University of Medical and Health Care, Hyogo, Japan; 4 Department of Physical Therapy, Sakai Heisei Hospital, Osaka, Japan; 5 Department of Physical Therapy, Tila Orthopedics Clinic, Okinawa, Japan; 6 Department of Physical Therapy, Kakogawa Central City Hospital, Hyogo, Japan; Gyeongsang National University, REPUBLIC OF KOREA

## Abstract

**Background:**

In archery training, side bridges are performed in a posture similar to archery shooting for training the muscles around the shoulder joint and the shoulder girdle of the pusher.

**Aim:**

The purpose of this study was to determine whether a low-tremor side-bridge exercise for 4 weeks improves bow tremor during archery movements.

**Methods:**

Participants were 20 male college students. First, we measured the tremor during side bridges performed with trunk inclinations of 25°, 40°, 55°, and 70° using an accelerometer attached to the elbow joint and identified low-tremor side bridges. The participants were then randomly divided into intervention and non-intervention groups, and the low-tremor side bridges were performed for 4 weeks.

**Results:**

The effect of the intervention was determined by measuring the total tremor value using an accelerometer attached to the bow and changes in the median power frequency (MdPF) of the middle deltoid, upper trapezius, and lower trapezius. This intervention reduced the bow tremor and the median power frequency of the middle deltoid (p < 0.05).

**Conclusions:**

The findings suggested that the tremor during the archery sighting phase could be reduced by performing side bridges with a specific trunk angle for a certain period of time. This intervention was also shown to reduce the intermediate frequency of the middle deltoid. The reduced tremor can shorten the sighting phase, which can facilitate injury prevention.

## Introduction

Archery is a sport enjoyed by millions of people of all ages and abilities worldwide, including people with physical mobility limitations in adaptive recreational or school physical education programs [1]. Various skills are necessary to achieve good performance in archery. Improvement in archery performance has been reported to be strongly related to spirit, skill, and fitness [2]. In addition, eye training, anxiety levels, and heart rate can influence the targeting accuracy [3–5]. Competitive archers make a large number of shots, and they are required to repeat the same action consistently [6, 7]. Thus, it is necessary to analyse various factors associated with archery skills that can improve archers’ performance. The precision-aiming task in archery is a static sport requiring strength and endurance of the shoulder girdle [8, 9]. In our previous study, we reported that the activities of the upper and lower trapezius muscles of the pull arm during archery shooting are associated with the development of trauma, which can influence the acquisition of archery skills but also cause conditions such as shoulder joint pain [10, 11]. Archery includes special movements; therefore, exercises with archery-specific characteristics are essential for individuals practicing this sport.

An archery shot primarily consists of three phases: 1) the stance phase; 2) the arming phase, during which the archer pulls the bowstring and pushes the bow; and 3) the sighting phase, which involves the final stretching of the bow while focusing on the target [6]. In the sighting phase, the archer needs to aim for a few seconds. Using electromyography (EMG), Leroyer et al. discovered a relationship between the ability level and the regularity of back muscle tremors during the final push-pull phase [6]. The bow and arm tremor in the sighting phase as well as the moment of the final release are important factors that influence this phase. Archers must keep their bows pulled with increased tension, leading to the appearance of bow and arm tremors, which can reduce archery performance. If the bow or arm tremors are large, the time required to aim in the sighting phase increases, and performance often drops owing to fatigue that is caused by extension of the sighting phase. Inappropriate shooting due to fatigue can not only reduce performance but also lead to trauma, such as shoulder pain. In other words, among competitive archers, exercises that reduce bow and arm tremor during the sighting phase are necessary to ensure optimal archery performance and prevent trauma.

In Japanese high schools, side-bridge exercise is performed to train the shoulder joint and shoulder girdle muscles of the pusher. This exercise resembles the shooting posture in archery and can be said to be an archery-specific exercise. Archers perform this exercise with their hands on the floor, and arm tremors often appear immediately after the start of the exercise. The tremor that appears during movement is associated with muscle activity and is caused by fatigue-related neural and mechanical mechanisms [12]. Muscle fatigue is associated with sustained and prolonged contractions, and is more associated with slow muscle fibre (type I) activity than fast muscle fibre (type II) activity [13, 14]. These findings indicate the importance of increasing the activity of slow muscle fibres of the muscles that are active in the shooting motion in order to reduce tremor in the sighting phase. Thus, appropriate activation of the slow muscle fibres during exercise is essential. Previous studies have reported a relationship between sports aptitude and training effects and muscle fibre composition [15–19] but there are no reports on its relationship with muscle fibre in archery-related exercise. The development of training that activates slow muscle fibers is desired by many archers.

To address this issue, we hypothesized that an analysis of arm tremors during side-bridge exercise performed under several levels of trunk inclination can reveal a threshold inclination that will exercise the slow muscle fibres associated with the tremor in archery, and that regular side-bridge exercise under these conditions will target the type of muscle fibres that are active during the sighting phase and reduce the tremor of the arm or bow. While this information is of inherent physiological interest, it can also improve the techniques currently employed in archery coaching.

Therefore, in this study, we first examined various side-bridge angles to determine the extent of trunk inclination that can be performed without the appearance of arm tremor during the side-bridge exercise. Then, we conducted a randomized controlled trial involving side-bridge exercise at the previously determined trunk inclination over a 4-week period to verify whether it improved bow tremor during archery movement. The preliminary hypotheses were that a larger trunk inclination angle during the side-bridge exercise would result in greater arm tremors, and that repetitive performance of the side-bridge exercise with a trunk inclination that did not cause tremors would reduce bow tremor during the sighting phase in archery.

## Materials and methods

### Participants

Twenty male collegiate students (20.8 ± 0.3, range: 20–23) participated in this study. All participants were inexperienced in archery. A completed and signed informed consent form was obtained from each participant before the study. The study protocol was approved by the Ethics Committee of the Takarazuka University of Medical and Health Care (No. 1805091), and all participants provided written informed consent. Participants were excluded if they had a history of dislocation of the shoulder, shoulder surgery, current symptoms related to the cervical spine, documented structural injuries to the shoulder complex, regular exercise habits, or hard training. Participants who reported any pain and/or discomfort in the upper extremities during the experiment were also excluded from the study. Participants were randomly assigned to the exercise and non-exercise groups using a lottery. Information on the participants in each group is presented in Table 1.

**Table 1:** Characteristics of the participants in this study.

	base line						
	intervention			non-intervention			p values
Age (years)	20.8	±	0.4	20.9	±	0.3	0.56
heigt (cm)	173.4	±	5.3	175.1	±	3.8	0.418
weight (kg)	62.1	±	4.7	65.7	±	5.8	0.142
BMI (kg/cm2)	20.7	±	1.7	21.5	±	2.4	0.403
tremor (×10^−4^)	8.68	±	4.90	7.69	±	5.24	0.225
deltoid middle MdPF (Hz)	76.27	±	13.64	71.96	±	9.83	0.712
trapezius upper MdPF (Hz)	72.42	±	16.19	65.82	±	11.75	0.711
trapezius lower MdPF (Hz)	64.03	±	13.69	59.74	±	7.68	0.680
serratas anterior MdPF (Hz)	51.83	±	9.93	47.91	±	10.05	0.684

### Archery task

**Fig 1:**
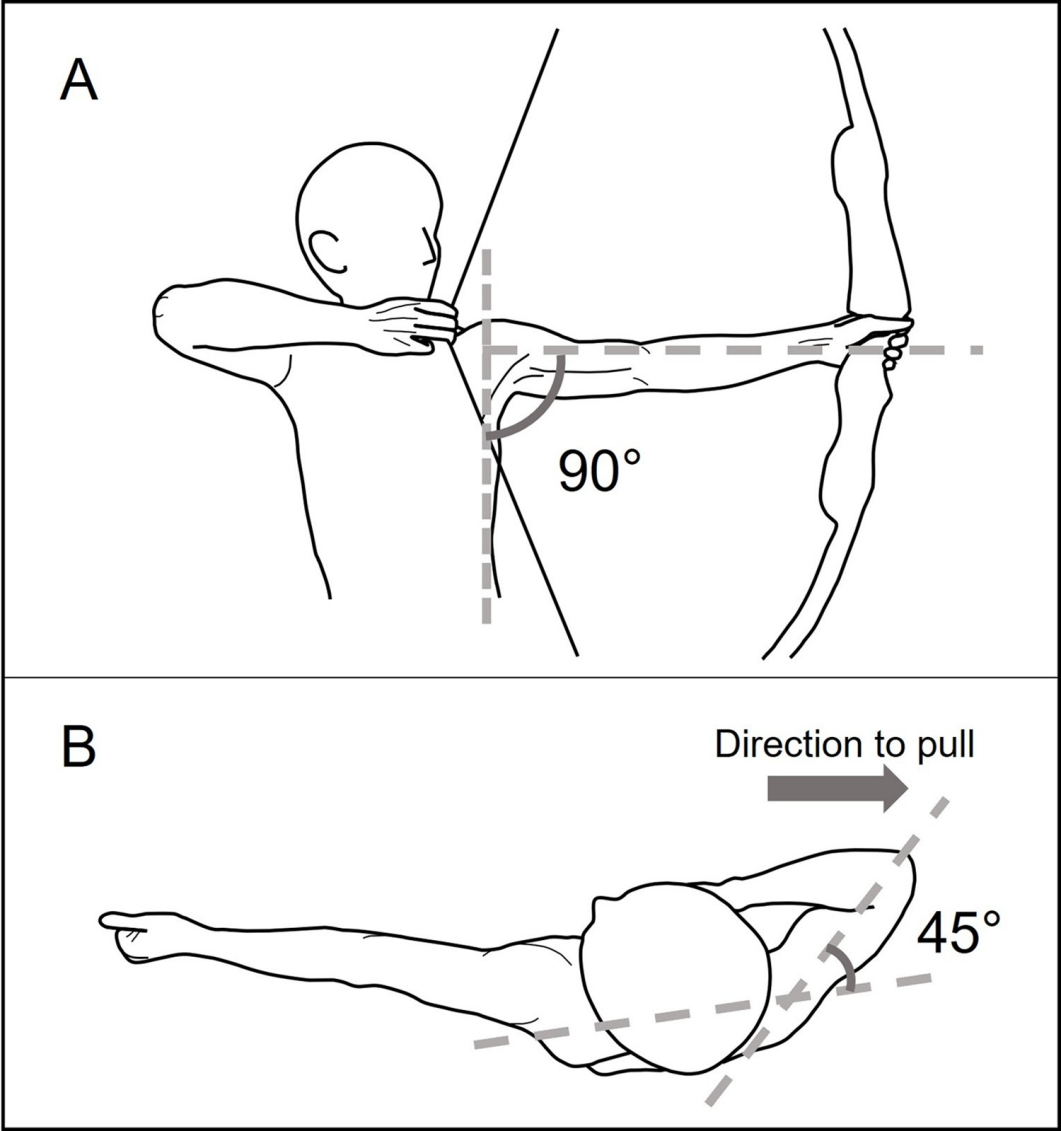
Posture during archery task. A is the frontal plane and B is the horizontal plane.

All 20 participants used a 9-kg (20 lb.) bow to perform archery shooting. The posture in this task was set to 90° abduction of bow-side shoulder joints (Fig 1A) and 45° horizontal flexion of draw-side shoulder joints (Fig 1B) with the upper left limb as the pusher and the upper right limb as the puller. Measurements were obtained in the sighting phase, and after confirming that the thumb of the puller was in contact with the chin, a signal was given and the sighting phase was performed for 15 s. Bow and arm tremor and the activities of the bow-side deltoid middle, upper trapezius, lower trapezius, and serratus anterior muscles were measured. Measurements for the same task were recorded again after 4 weeks in both the intervention and non-intervention groups.

### Selection of trunk inclination during the side-bridge exercise

**Fig 2:**
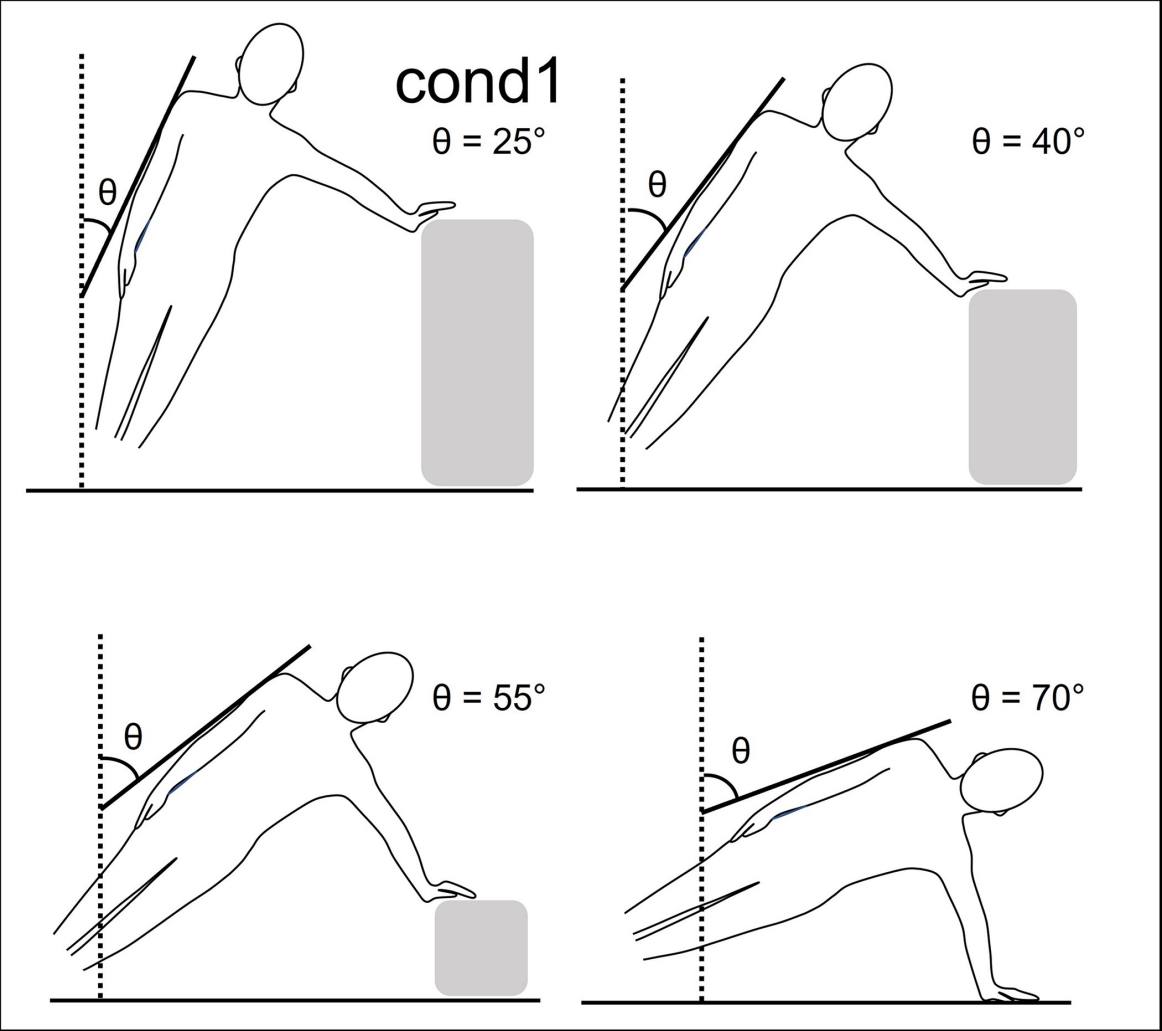
Four conditions used in the side-bridge exercise. Cond1, cond2, cond3, and cond4 represent trunk inclinations of 25°, 40°, 55°, and 70°, respectively.

Eleven participants were included in this study. The side bridge exercise was performed for 15 seconds in each of the four conditions and arm tremor was analysed. Since all participants were right-handed, they performed the exercise with their left hand holding a bow during archery shooting. For all side-bridge exercises, the bow-side shoulder joint was abducted at 90°, and the elbow joint was extended at 0°. The four side-bridge conditions involved trunk inclinations of 25° (Fig 2, cond1), 40° (Fig 2, cond2), 55° (Fig 2, cond3), and 70° (Fig 2, cond4), and a stand was used to fix the inclination. A non-slip mat was used for the floor and the stand. The subjects rested between each condition.

### Exercise intervention

**Fig 3:**
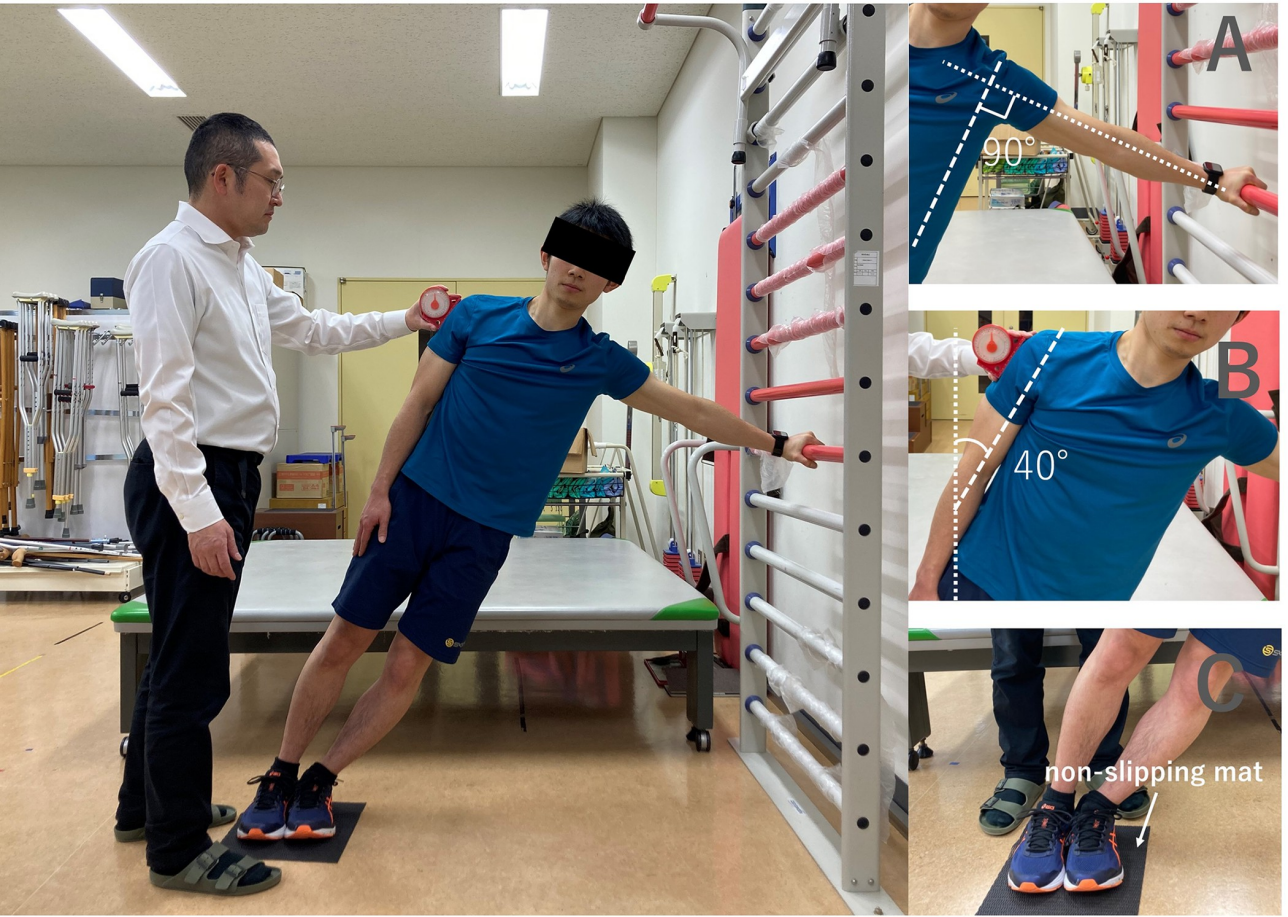
The side-bridge exercise performed in the intervention. The height of the Bow-side hand was adjusted and the trunk inclination angle was 40 degrees. A non-slip mat was used for the floor and the stand.

The intervention group exercised 5 days a week for 4 weeks, for a total of 20 days. The exercise was a side bridge performed with a trunk inclination of 40°, which was judged to be the most effective of the four side-bridge exercises (Figs 2B, 3). When performing the exercise, the height of the table should be adjusted with the shoulder joint in 90-degree abduction. An angle meter was placed on the upper arm of the draw-side to check the trunk inclination (Fig 3). The participants performed five 1-min sets of exercise with 1 min of rest after each set, resulting in a total of approximately 10 min of exercise a day. The exercise was performed in a laboratory at the university, and the exercise environment was unified throughout the study period. Although the time of exercise was not pre-decided, we confirmed by e-mail that the participants had completed the exercise each time.

### Data analysis

#### Analysis of tremor

For the Bow tremor measurement, the accelerometers were fixed to the handle with taping. To measure arm tremor, the accelerometers were fixed to the lateral epicondyle of the humerus with taping. Bow and arm tremor were measured using accelerometers (OE-WS2521; Oisaka, Hiroshima, Japan) fixed to the handle of the bow by taping. This device is an integrated accelerometer and surface EMG sensor that is also used to measure muscle activity.

The accelerometer and EMG data obtained in this study were analyzed using Python (version: 3.10.6, packaged by conda-forge), pandas (version: 1.4.3), numpy (version: 1.22.4), matplotlib (version: 3.5.3), scipy.stats (version: 1.9.0), and scikit_posthocs (version: 0.7.0). The sampling frequency was 1 kHz, the sampling time was 15 seconds, and the obtained acceleration data (*a*_*x*_, *a*_*y*_, *a*_*z*_) were synthesized.


$$acceleration=\sqrt{a_{x}^{2}+a_{y}^{2}+a_{z}^{2}}$$ (1)

If the measurement time is *t* and the sampling frequency is *f*, the formula for the data sample size n is (2). Since the measurement time of this study is 15 seconds and the sampling frequency is 1000 Hz, the measured sample size is 15,000.


n=tf (2)

Since the Fast Fourier Transform algorithm to be used later includes butterfly operations, the sample size to be analyzed should satisfy (3) when the natural number is N. The sample size for analysis is smaller than the measured sample size. In other words (4), which must be smaller than the measured sample size (15000). In the case of this study, the maximum value satisfying (3) and (4) is 8192 (= 2^13^). Data were excluded from 6808, which is the measured sample minus the set sampling size, for each of the initial and last 3.404 seconds.


$$n=\{2^{x}|x\in N\}$$ (3)

n≤tf (4)

The data for the intermediate 8.192 s were converted into frequency data using a fast Fourier transform (FFT) (5, 6); and the power spectrum was calculated. The sum of the values in the 3–18-Hz frequency band was analysed as the tremor value. The programming code used for the analysis is attached in the (S1 Text (Selection of trunk inclination during the side-bridge exercise)). The data used in the analysis can be found below (https://data.mendeley.com/datasets/28hzmcs9vy/1).


$$F(k)=\sum_{x=0}^{n-1}f(x)e^{-j\frac{2\pi k}{n}x}$$ (5)

$$\Phi (k)={\left|F(k)\right|}^{2}$$ (6)

#### Muscle activity analysis

Bipolar surface electrodes (OE-WS2521; Oisaka, Hiroshima, Japan) were placed on three scapulothoracic muscles (the upper and lower trapezius and the serratus anterior) and a scapulohumeral muscle (deltoid middle) in accordance with previously published standardized methods [20] and inter-electrode distance was 20 mm. EMG data underwent analogue/digital (A/D) conversion at 1 kHz (LabVIEW, National Instruments). Of the obtained 15-s myoelectric data, the data for the initial and final 3.404 s were deleted; the power spectrum was calculated by FFT analysis of the data for the remaining 8.192 s; and the median power frequency (MdPF) was calculated [21].


$$\sum_{k=0.001}^{f_{mdpf}}F(k)=\sum_{k=f_{mdpf}}^{\infty }F(k)=\frac{1}{2}\sum_{k=0,001}^{\infty }F(k)$$ (7)

The frequency band for the analysis was 1–1000 Hz. Since the measured values are affected by electrode position and skin temperature, photographs of the target site were taken before and after the intervention, and the measurements were performed at the same sites, with the measurements for all muscles obtained simultaneously [22, 23]. The programming code used for the analysis is attached in the (S2 Text (Exercise intervention)). The data used in the analysis can be found below (https://data.mendeley.com/datasets/28hzmcs9vy/1).

### Statistical analysis

Statistical analysis was performed using the Statistical Package for the Social Sciences (SPSS Inc., Chicago, IL, USA) version 20.0 J for Windows®. The Steel-Dwass method was used for statistical evaluations. The baseline values of the intervention and the non-intervention groups were compared using Welch’s t-test. A paired t-test was performed before and after the training intervention. The effect size (d) was calculated from the baseline and the values after 4 weeks in the intervention and non-intervention groups. The probability level accepted as the criterion for statistical significance was set at 5%.

## Results

### Verification of optimal trunk inclination at the time of the side-bridge exercise

**Fig 4:**
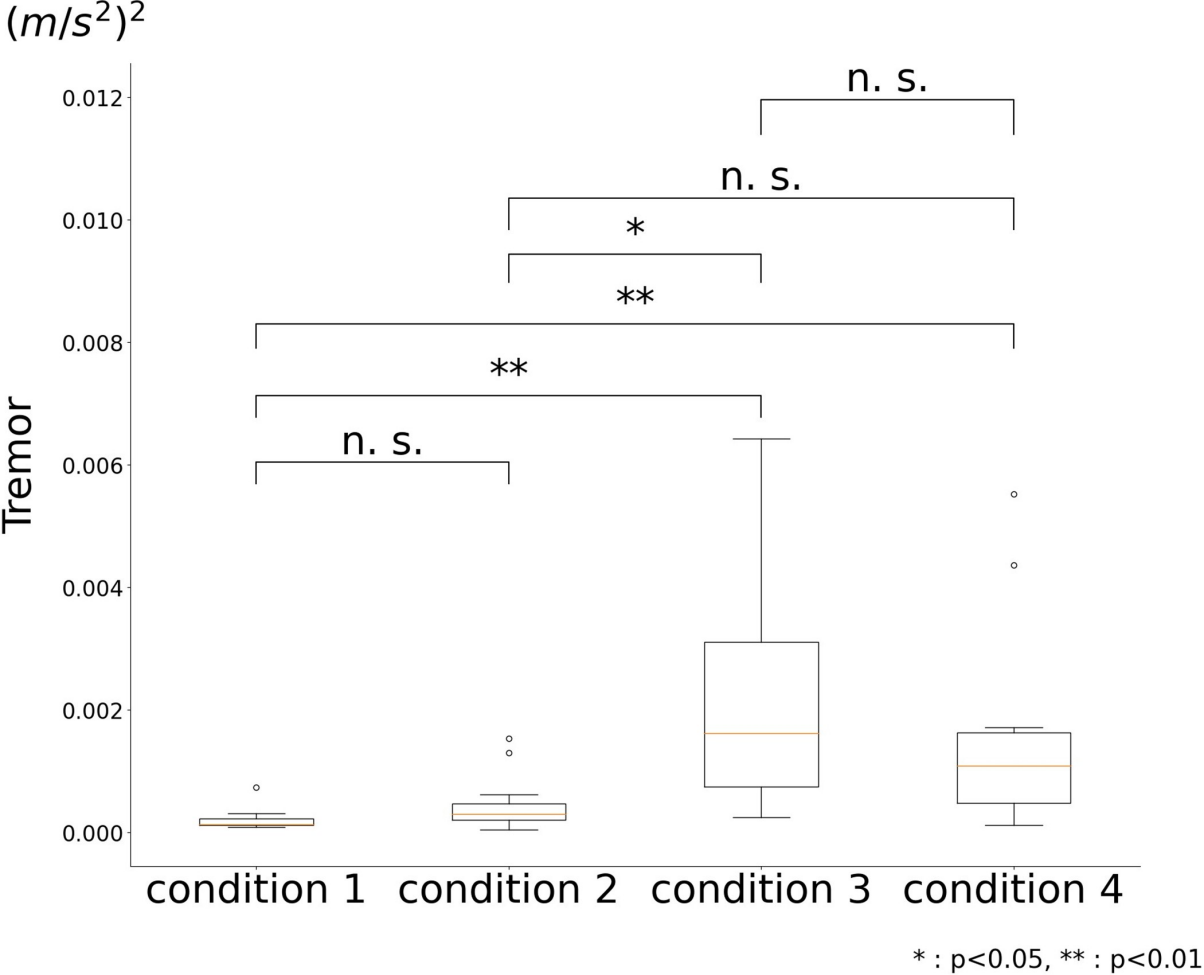
Comparison of tremors during side-bridge exercise.

The arm tremor values were 0.00022 ± 0.00018(m/s^2^)^2^ for cond1, 0.00048 ± 0.00047(m/s^2^)^2^ for cond2, 0.00215 ± 0.00182(m/s^2^)^2^ for cond3, and 0.00161 ± 0.00167(m/s^2^)^2^ for cond4. A Kruskal–Wallis test for the four conditions yielded p-values less than 5%. Therefore, a multiple-comparison test was performed, and it showed that the total arm tremor value during exercise was not significantly different between the 25° and 40° conditions, but was significantly different among the 25° (or 40°), 55°, and 70° conditions (Fig 4). Thus, the results suggested that tremors occurred at trunk inclinations of 55° or more, and the side-bridge with 40° trunk inclination (cond2) was used as the intervention exercise.

### Comparison of baseline values between the intervention and non-intervention groups

The baseline age, height, body weight, and BMI were not significantly different between the intervention and non-intervention groups. The two groups also showed no significant differences in baseline tremor intensity and MdPF values for the middle deltoid, upper trapezius, lower trapezius, and serratus anterior muscles (Table 1).

### Comparison of values before and after the intervention

Measurements obtained during shooting movements at baseline and after 4 weeks in the intervention group were compared, and significant differences were observed in the intensity of tremors and the MdPF of the middle deltoid (Table 2). However, no significant intervention-related differences were observed in the MdPF of the upper trapezius, lower trapezius, and serratus anterior muscles. In addition, none of the items in the non-intervention group showed significant differences in the findings obtained at the two evaluation points (Table 3).

**Table 2:** Comparison between the findings at baseline and 4 weeks in the intervention group.

	base line (mean±SD)			after 4 weeks (mean±SD)			p values	effect size (d)	base line (min-max)			after 4 weeks (min-max)		
tremor (×10^−4^) (m/s^2^)^2^	8.68	±	4.90	6.83	±	4.84	0.047	0.72	1.34	–	17.70	0.98	–	17.77
deltoid middle MdPF (Hz)	76.27	±	13.64	68.04	±	9.92	0.003	1.30	48.83	–	99.13	53.60	–	85.58
trapezius upper MdPF (Hz)	72.42	±	16.19	71.37	±	13.11	0.850	0.06	51.28	–	99.87	54.21	–	92.30
trapezius lower MdPF (Hz)	64.03	±	13.69	58.14	±	6.73	0.076	0.63	44.19	–	91.81	47.74	–	67.27
serratas anterior MdPF (Hz)	51.83	±	9.93	53.78	±	13.25	0.439	0.25	32.35	–	66.41	26.98	–	74.84

**Table 3:** Comparison between the findings at baseline and 4 weeks in the non-intervention group.

non-intervention group														
	base line (mean±SD)			after 4 weeks (mean±SD)			p values	effect size (d)	base line (min-max)			after 4 weeks (min-max)		
tremor (×10^−4^) (m/s^2^)^2^	7.69	±	5.24	8.19	±	9.09	0.854	0.06	1.71	–	18.74	0.69	–	31.75
deltoid middle MdPF (Hz)	71.96	±	9.83	69.21	±	10.13	0.233	0.40	52.74	–	89.24	51.15	–	86.56
trapezius upper MdPF (Hz)	65.82	±	11.75	72.07	±	15.25	0.311	0.34	49.08	–	86.07	47.86	–	99.74
trapezius lower MdPF (Hz)	59.74	±	7.68	56.96	±	10.26	0.320	0.33	45.29	–	71.66	42.73	–	73.74
serratas anterior MdPF (Hz)	47.91	±	10.05	50.23	±	8.37	0.528	0.21	24.05	–	61.16	41.88	–	68.73

## Discussion

In the arming phase of archery shooting, fast muscle fibre activity is required to pull the strings strongly, and in the sighting phase, the target must be aimed while keeping the strings pulled out. In this study, we hypothesized that the activity of slow muscle fibres was necessary to reduce bow tremor in the sighting phase and identified an exercise and verified its effect by performing randomized controlled trials. A comparison of the exercise methods under the four conditions showed that the side bridge exercise, in which the trunk is tilted 40°, is the condition with the least tremor and the greatest trunk inclination. Therefore, we hypothesized that a side bridge at a 40° inclination could be performed with less tremor in the sighting phase and examined the effect of 4-week training with this exercise on tremor.

The results suggested that exercises aimed at reducing tremors during the shooting action in archery are effective when the exercise itself is performed with a load that does not cause tremors to appear. The participants also showed a reduction in the MdPF of the middle deltoid after 4 weeks of exercise. Thus, side-bridge exercise performed with a trunk angle adjusted to avoid the appearance of tremors appeared to reduce the tremor in the sighting phase of archery and also changed the activity of the middle deltoid muscle. The activity of motor units that control slow muscle fibres has been shown to mainly reflect low-frequency components, while that of fast-muscle fibres reflects high-frequency components [24, 25]. Thus, for the middle deltoid muscle, the activity rate of slow muscle fibres appeared to increase with exercise.

Previous studies have shown that nervous system factors change up to 4 weeks after the commencement of exercise, and that muscle hypertrophy causes muscle strengthening 8 weeks after starting the exercise [26]. Therefore, if the tremor reduction observed in this study was affected by muscle activity, it may have been mediated by an increase in the number of motor units, increased and more synchronized frequency of firing, decreased activity of antagonist muscles, and improved coordination with joint muscles. These findings may have occurred in the slow muscle fibres. In addition, since the exercise itself used almost the same posture as the outcome, it may have involved a motor-learning effect allowing the participants a sense of "getting used to the posture." In previous studies, this precision-aiming task was specified as a static sport requiring strength and endurance of the shoulder girdle [8, 9]. This study showed that continuous muscle contraction without tremor enhances the activity of slow muscle fibres and affects archery performance.

Tremors can be divided into rest and action tremors based on their occurrence, and tremors that occur during exercise can be classified into postural, isometric, and kinetic tremors [27, 28]. The tremors appearing in the sighting phase of archery shooting motion are predicted to be postural and isometric tremors. In previous studies, the activity of the shoulder joint and the muscles around the shoulder girdle in the shooting motion and the shoulder joint angle were shown to be related to tremors of the scapula and humerus. Furthermore, the activity of the muscles around the shoulder girdle was shown to be important for achieving stability. In that study, the measured frequency bands were 3–7 Hz and 8–12 Hz in consideration of the stretch reflex and supraspinal systems [29]. Since no previous report has described the frequency band of tremors that affect performance in archery shooting, this study used the frequency bands reported in diseases manifesting with postural tremors, including Parkinson’s disease (4–6 Hz) [30], essential tremors (4–12 Hz) [31], orthostatic tremors (13–18 Hz) [32], and neuropathic tremors (3–6 Hz) [33]. On the basis of the assumption that tremors across these frequency bands may affect motion, a frequency band of 3–18 Hz was analysed in this study.

The exercise performed in this study can be performed with 90° abduction of the shoulder joint, which is the same as the archery target posture, and the angle of the side-bridge exercise is defined from the trunk inclination, making it a completely new exercise unlike the previously performed side bridge. In addition, the exercise duration was set to 1 min per set, and five sets were performed. In previous studies, male college students were reported to perform a side bridge for an average period of 97 s, and in other previous studies, the exercise was performed as long as possible [34, 35]. However, this study aimed to identify an exercise intensity that could be performed without tremor and was not aimed at all-out exercise. It is also unclear whether the four-week intervention period was appropriate. Most of the study participants did not have regular exercise habits; thus, even a short exercise may have been effective in showing results in these participants.

The limitations of this study are as follows. First, the participants were not professional archers; therefore, the effectiveness of the intervention exercise on the actual shooting movements of professional archers was unclear. However, to verify the effect of an exercise in one sport, it is desirable to assess the exercise in participants who do not perform other sports, and the present study met this criterion. Second, the side-bridge exercise assessed in this study was performed at a trunk angle at which tremor did not occur, but it was unclear which of the other tremor-inducing conditions was the most effective in reducing tremors. Among the exercise conditions evaluated in this study, cond4 can be excluded because it is difficult to perform and it is difficult to set the exercise time for this condition. Third, the effect of the exercise was judged solely on the basis of the information obtained by the accelerometer and the electromyogram, and the related physiological changes are unknown. The muscles evaluated in this study were the four muscles around the shoulder joint. Since side-bridge exercise is also used for core muscle training, other muscle groups may have influenced the results of this study, but their influence could not be determined from the results of this study.

## Conclusions

The findings of this study may provide an effective training method for athletes who cannot control their tremors during shooting movements, such as beginner archers and those experiencing yips. In future research, we would like to verify the appropriate load, such as the angle of the trunk and exercise time for actual competitive archers, and to verify the effect of the intervention in improving performance and preventing trauma.

## Supporting information

S1 Text Selection of trunk inclination during the side-bridge exercise. The code was written using Python.
(TXT)

S2 Text Exercise intervention. The code was written using Python.
(TXT)
